# The role of pneumolysin in mediating lung damage in a lethal pneumococcal pneumonia murine model

**DOI:** 10.1186/1465-9921-8-3

**Published:** 2007-01-26

**Authors:** María del Mar García-Suárez, Noelia Flórez, Aurora Astudillo, Fernando Vázquez, Roberto Villaverde, Kevin Fabrizio, Liise-Anne Pirofski, Francisco J Méndez

**Affiliations:** 1 Área de Microbiología, Departamento de Biología Funcional, Instituto Universitario de Biotecnología de Asturias (IUBA), Universidad de Oviedo; 33006 Oviedo, Asturias, Spain; 2 Laboratorio de Anatomía Patológica, Instituto Universitario de Oncología del Principado de Asturias (IUOPA), Universidad de Oviedo; 33006 Oviedo, Asturias, Spain; 3 Department of Microbiology and Immunology, Albert Einstein College of Medicine, 1300 Morris Park Avenue, Bronx, New York 10461, USA; 4 Division of Infectious Diseases, Department of Medicine, Albert Einstein College of Medicine and Montefiore Medical Center, 1300 Morris Park Avenue, Bronx, New York 10461, USA

## Abstract

**Background:**

Intranasal inoculation of *Streptococcus pneumoniae *D39 serotype 2 causes fatal pneumonia in mice. The cytotoxic and inflammatory properties of pneumolysin (PLY) have been implicated in the pathogenesis of pneumococcal pneumonia.

**Methods:**

To examine the role of PLY in this experimental model we performed ELISA assays for PLY quantification. The distribution patterns of PLY and apoptosis were established by immunohistochemical detection of PLY, caspase-9 activity and TUNEL assay on tissue sections from mice lungs at various times, and the results were quantified with image analysis. Inflammatory and apoptotic cells were also quantified on lung tissue sections from antibody treated mice.

**Results:**

In bronchoalveolar lavages (BAL), total PLY was found at sublytic concentrations which were located in alveolar macrophages and leukocytes. The bronchoalveolar epithelium was PLY-positive, while the vascular endothelium was not PLY reactive. The pattern and extension of cellular apoptosis was similar. Anti-PLY antibody treatment decreased the lung damage and the number of apoptotic and inflammatory cells in lung tissues.

**Conclusion:**

The data strongly suggest that *in vivo *lung injury could be due to the pro-apoptotic and pro-inflammatory activity of PLY, rather than its cytotoxic activity. PLY at sublytic concentrations induces lethal inflammation in lung tissues and is involved in host cell apoptosis, whose effects are important to pathogen survival.

## Background

*Streptococcus pneumoniae *is the major pathogen of community-acquired pneumonia and one of the most common causes of death due to infectious disease in industrialized countries. Pneumococcus usually colonizes the nasopharynx of humans asymptomatically, although on occasions it passes from this niche to the lungs, brain, and blood [1,2]. This can lead to diseases associated with high morbidity and mortality such as pneumonia, septicemia, and meningitis. Pneumolysin (PLY) is a 53-kDa toxic protein that belongs to the family of antigenically related thiol-activated, cholesterol-binding cytolysins [3]. At high levels, PLY is lytic to all cells with cholesterol-containing membranes [4]. In contrast to other characterized cytolysins, it is located in the cytoplasm and released during bacterial growth and lysis [5]. PLY contributes to disease mortality, and mutants of the *ply *gene have reduced virulence in mice after pulmonary challenge [6-8]. PLY has proven to be a protective immunogen in mice [9,10] against challenge with a range of capsular serotypes [11]. As such, PLY is considered to be an excellent candidate to include in a pneumococcal vaccine [1,12].

Pneumococci are capable of inducing apoptosis in respiratory tree epithelium [13,14], endothelium, and neuronal cells [15,16]. *S. pneumoniae *produces two morphologically distinct forms of programmed cell death [15]. The apoptotic activity of PLY in dendritic and cerebral endothelial cells is caspase-independent [15,17,18]. Caspase-dependent and TLR-4-mediated apoptosis is elicited by *S. pneumoniae *serotype 3 in nasopharyngeal epithelium in a murine model of nasal colonization [14]. Microbe-induced apoptosis may represent a major mechanism by which pathogenic bacteria avoid detection and destruction by the innate immune system [19]. Certain pathogens use virulence factors to dismantle host defenses through inhibition of anti-apoptotic signaling pathways [20,21]. PLY induces apoptosis [18,22], activates complement [23], and releases proinflammatory mediators [24,25]. In this study, we examined the role of PLY in mediating lung damage in experimental acute bacterial pneumonia induced by *S. pneumoniae *D39 serotype 2.

## Methods

### Murine infection

Mice were intranasally inoculated as previously described [26]. Briefly, outbred MF-1 mice (Oxon, Harland Olac Ltd., Bicester, England) weighing 30 ± 3 g were lightly anaesthetized with 3% (v/v) halothane over oxygen (3–4 l/min) using a methacrylate box connected to Fluovac 240 (Anaesthetizing system, Cheshire, England) and intranasally infected with a lethal dose of 5 × 10^6 ^CFU of *S. pneumoniae *D39 serotype 2 NCTC 7466 (Spanish Type Culture Collection, Valencia, Spain) in 50 μl of phosphate-buffered saline (PBS), applied atraumatically to the tip of the nose and involuntarily inhaled. Animal studies were performed in accordance with the guidelines of the Institutional Animal Care and Use Committee of the University of Oviedo (Spain).

### Bronchoalveolar lavages (BAL)

Groups of 3 mice were deeply anaesthetized 12, 24, 36, 48, 60 and 72 h after infection. The trachea was surgically exposed and cannulated. BAL was performed by a single injection of 0.5 ml of PBS into the trachea, followed by aspiration through a 25-G needle. Quantitative cultures from BAL were then performed on blood agar to determine the number of colony-forming units (CFU).

### PLY detection by ELISA

Quantification of PLY was performed by ultrasensitive enzyme-linked immunoassay (ELISA) as described previously [27]. Briefly, Triton X-100 to 0.05% was added to the BAL samples and incubated 30 min at 37°C to allow pneumococcal lysis. Flat-bottomed polystyrene Combiplate White Breakable (Labsystems, Helsinki, Finland) plates were coated with 1 μg/well of PLY-7 (IgG1 kappa, anti-PLY mouse monoclonal antibody) in carbonate-bicarbonate buffer 0.05 M pH 9.6 for 6 h at 37°C. Plates were washed at each step with PBS plus 0.1% Tween-20, blocked with blocking buffer prepared according to the instructions of the manufacturer of ELISA-Light™ Chemiluminescent Detection System (Tropix, Applied Biosystems, Bedford, MA, USA). The samples were then added to wells and incubated at 37°C for 1 h with shaking. Once washed six times, plates were incubated with rabbit IgG polyclonal anti-PLY diluted in blocking buffer and incubated 30 min at 37°C. An alkaline phosphatase conjugated goat anti-rabbit IgG secondary antibody (Sigma Chemicals Co.) was used at a 1:5000 dilution and incubated as above. Plates were loaded on a Luminoskan RS (Labsystems) luminometer and the wells were automatically filled with substrate/enhancer solution (0.4 mM CSPDR with 1× Sapphire-II™) and incubated for 10 min. The lower detection limit of ELISA assay was 30 pg/ml of PLY.

### Antibody treatment

Mice intranasally infected with *S. pneumoniae *D39 serotype 2 were treated with anti-PLY rabbit IgG as previously described [26]. Briefly, mice were injected in the tail vein with 100 μg of anti-PLY IgG [26] in 200 μl of sterile non-pyrogenic PBS 1 h before and 36 h after intranasal infection with *S. pneumoniae*. Control mice were injected with 100 μg of non-immune rabbit IgG (Sigma) or 200 μl of sterile non-pyrogenic PBS. Groups of mice were deeply anaesthetized 12, 24, 36, 48, 60 and 72 h after infection, and lungs were removed, fixed in 10% buffered formalin, and embedded in paraffin.

### Histopathology and immunohistochemistry

For confocal examinations, 5 μm sections were washed in fresh xylene for 5 min, rehydrated through a series of graded alcohols and air dried at room temperature. 50 μl of SYTO 9 green fluorescent nucleic acid stain (LIVE/DEAD Bac-Light Bacterial Viability Kit, Molecular Probes, L-13152) were added to tissue sections, and the cover-slide was placed on top after staining for at least 10 minutes in the dark. The samples were then examined by Z stacking under a Leica TCS-SP2-AOBS confocal laser scanning microscope at a wavelength of 488 nm excitation and 530 nm (green) emission. Images were captured using the Leica Confocal Software. For histology examinations, sections were stained with hematoxylin and eosin (H&E) and viewed by light microscopy. For immunostaining, sections mounted on slides were baked for 30 min at 60°C and then washed twice in fresh xylene for 5 min each to remove paraffin. The slides were rehydrated through a series of graded alcohols, and washed in distilled water for 3 min. Endogenous peroxidase activity was blocked using a peroxidase-blocking solution (DAKO, Glostrup, Denmark) and non-specific binding was blocked with 1% bovine serum albumin (BSA) in Tris-buffered saline (TBS) (100 mM Tris, pH 8.0; 150 mM NaCl). After antigen retrieval, lung tissue sections were incubated with rabbit polyclonal anti-PLY IgG [26,27] diluted to 1:1000 in 1% BSA-TBS for 16 h at 4°C and visualized using the DAKO EnVision™ +Kit (DAKO). For caspase-9 detection, lung tissue sections were incubated with rabbit anti-caspase-9 mouse specific antibody (Cell Signaling Technology Inc., Beverly, MA, USA) diluted to 1:100 in 1% BSA-TBS for 16 h at 4°C, followed by washing in TBS-0.1% Tween-20, and visualized as above. The TUNEL assays of tissue sections were conducted using the *In Situ *Cell Death Detection Kit, POD (Roche Applied Science, Penzberg, Germany) following manufacturer's instructions. Sections were washed and counterstained briefly with hematoxylin. Four sections separated by at least 200 μm were studied per animal and examined using a light microscope Leica DMR (Leica Microsystems Wetzlar GmbH, Germany) coupled to a high resolution colour Leica MPS30 camera. Analysis was carried out with the UTHSCSA Image Tool for Windows Version 3.0 software programme (University of Texas Health Science Center, San Antonio, TX, USA). Tissue areas were selected using systematic random sampling and cells were counted in five areas delineated by a grid. For co-localization of PLY, TUNEL, and caspase-9, three adjacent sections were co-stained. Thereafter, we acquired images and identified matching cells in the sections by overlaying the PLY immunostaining. A total of five sections were analyzed for each time point.

### Statistical analysis

Statistical differences in total PLY amounts at different time points were analyzed by the nonparametric Mann-Whitney *U *test. Correlation between PLY and CFU was performed by nonparametric Spearman *r*-test. Statistical differences in the percentage of positive cells of PLY-, TUNEL- and caspase-9-staining, percentage of caspase-9 positive cells, and numbers of infiltrating cells among treatment groups were calculated by two-way ANOVA followed by the Bonferroni test. All statistical analyses were performed using Prism (v.4.00 for Windows; GraphPad Software, San Diego, CA). The limit of statistical significance was a *P *value of 0.05.

## Results

### PLY quantification and pneumococci localization

**Figure 1:**
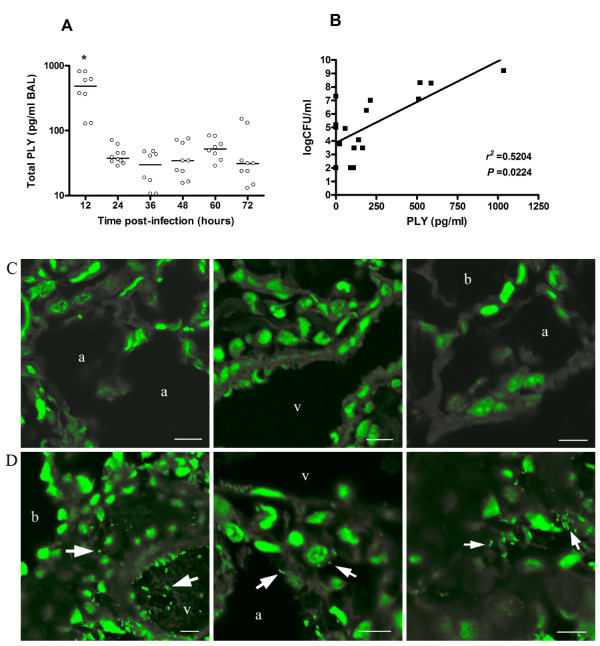
**Concentrations of PLY and bacteria localization in lungs of mice infected with *S. pneumoniae *D39 serotype 2**. (A) Amounts of total PLY were quantified in BAL after lysis of bacteria. Each symbol represents one mouse, and horizontal bars represent medians. Results are representative of three independent experiments. Groups were compared by nonparametric Mann-Whitney *U *test. * *P *< 0.05. (B) Correlation between CFU and PLY in BAL. Dots represent the means of CFU versus PLY concentration from three mice at the same time points of Fig. 1A Correlation was performed by nonparametric Spearman *r*-test. (C) Confocal images of lung tissue sections from uninfected mice. (D) Representative lung tissue sections from pneumococci infected mice showing intra-vessel, intra-cytoplasmic and intercellular bacteria localization. Blood vessel (v), alveolar space (a), bronchiole (b). Scale bars 8 μm. All images were captured after a Z-stack analysis of the samples.

Quantification of PLY was performed in bronchoalveolar lavages (BAL) obtained at different time points during pneumococcal pneumonia from mice infected intranasally with *S. pneumoniae *D39 serotype 2 (Figure 1A). PLY was undetectable in BAL samples after removal of bacteria by centrifugation. In contrast, PLY was detected after lysis of bacteria, showing the highest level at 12 h post-infection (approximately 1000 pg/ml) compared with other time points (*P *< 0.05). A positive correlation was found between concentrations of total PLY and number of bacteria present in BAL (*r*^2 ^= 0.5204, *P *= 0.0224) (Figure 1B). To investigate whether changes in PLY amounts during pneumonia were associated with different localizations of bacteria in the lungs, an examination of tissue samples was performed by confocal microscopy.

Pneumococcal DNA was stained and bacteria were recognized as diplococcal forms, which were not present in uninfected lung tissues (Figure 1C). Analysis of the z-stacks obtained on the confocal microscope revealed that both intra-and intercellular pneumococci were found in infiltration areas, and after 24 h post-infection pneumococci were observed inside capillary vessels (Figure 1D). Bacteria were not observed inside endothelial or epithelial cells.

### PLY and apoptosis localization

**Figure 2:**
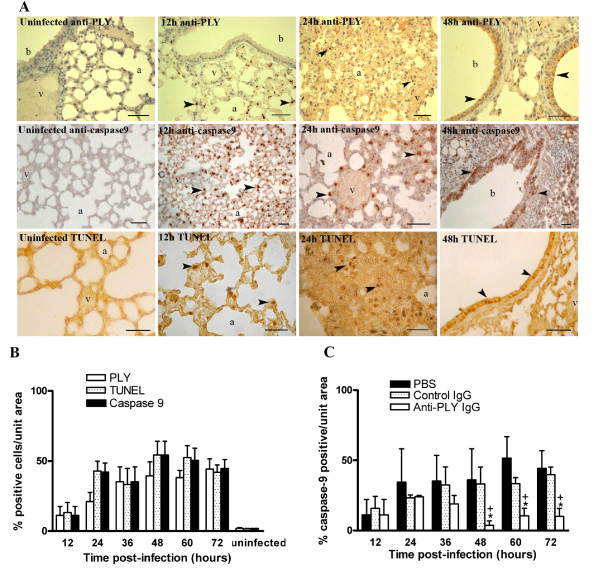
**Apoptosis in lung tissues of mice infected with *S. pneumoniae *D39 serotype 2**. (A) Distribution patterns of PLY and apoptosis in representative lung sections from mice intranasally infected with *S. pneumoniae *D39 serotype 2. PLY was established by staining with anti-PLY rabbit antibodies. Apoptosis was assessed by active caspase-9 staining and *in situ *TUNEL assay. No staining was observed in lung tissues from uninfected mice. At 12 h post-infection, resident alveolar macrophages were positively stained with anti-PLY, anti-caspase-9, and TUNEL (arrow heads). Infiltrating leukocytes (24 h post-infection) and bronchial epithelium (48 h post-infection) were stained with anti-PLY, anti-caspase-9, and TUNEL, respectively (arrow heads). Note non-stained vascular endothelium. Blood vessel (v), alveolar space (a), bronchiole (b). Scale bars 50 μm. (B) Apoptosis and PLY in lung tissues from untreated mice during pneumococcal pneumonia. Apoptosis was identified by immunohistochemical detection of active caspase-9 and by *in situ *TUNEL assay. PLY was stained with anti-PLY rabbit antibodies. Adjacent sections were co-stained for co-localization of PLY, TUNEL, and caspase-9. Five sections were analyzed in each time point. Statistical differences were not found for a comparison of number of PLY, caspase-9, and TUNEL positive cells as determined by two-way ANOVA followed by the Bonferroni test. (C) Comparison of caspase-9 positive cells in lung tissues from anti-PLY IgG-, control IgG-, and PBS-treated mice. Percentage of caspase-9 stained cells was calculated with respect to total cells counted in random areas of lung tissue sections. Results are means ± SD of 3 mice and are representative of three independent experiments. *, *P *< 0.05 for a comparison of anti-PLY IgG-treated mice with PBS-treated mice, and +,*P *< 0.05 for a comparison of anti-PLY IgG with control IgG-treated mice, as determined by two-way ANOVA followed by the Bonferroni test.

PLY localization was performed by specific immunostaining in lung tissues from mice during progression of experimental pneumococcal pneumonia. At 12 h post-infection, PLY staining was detected in resident alveolar macrophages (Figure 2A). After 24 h post-infection, leukocytes located in perivascular and peribronchial infiltration areas and bronchial epithelium showed PLY-stain. Vascular endothelium was PLY-stained at no time during pneumococcal infection. No staining was observed in lungs from non-infected mice.

Apoptosis localization in lung tissues during pneumococcal pneumonia was performed by *in situ*-TUNEL assay and by specific immunostaining of active caspase-9. TUNEL- and caspase-9-staining were located in alveolar macrophages at 12 h post-infection (Figure 2A). Leukocytes situated in areas of cellular infiltration, and bronchial epithelia appeared progressively stained after 24 h post-infection. Neither TUNEL nor caspase-9 staining was found in vascular endothelium. Apoptosis staining was not observed in lungs from non-infected mice.

Because the anti-PLY and anti-caspase-9 antisera available for immunohistochemistry had been raised in rabbits, we could not perform double staining on the same tissue section. For co-localization of PLY, TUNEL, and caspase-9, three adjacent sections were co-stained. Counting the number of positive cells per unit area in consecutive sections, it was shown that there were no statistical differences in the number of cells staining for PLY, TUNEL, and caspase-9 (*P *> 0.05) (Figure 2B).

To determine the pro-apoptotic activity of PLY, the number of caspase-9 stained cells was compared in lung tissue sections obtained at different times during pneumococcal pneumonia from PBS-, control IgG-, and anti-PLY IgG-treated mice. Lung tissue sections from anti-PLY IgG-treated mice have a lower percentage of caspase-9 stained cells than PBS- (*P *< 0.01) and control IgG-treated mice (*P *< 0.05), at 48 h, 60 h and 72 h post-infection (Figure 2C). Analogous results were obtained from the number of TUNEL stained cells (data not shown).

### Leukocyte recruitment

**Figure 3:**
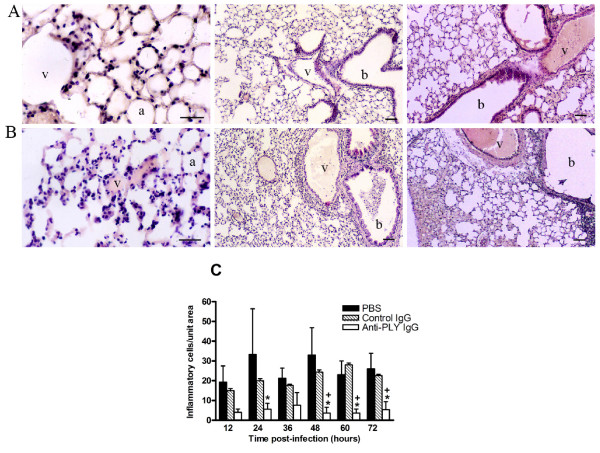
**Comparison of the level of inflammation of lung tissue among anti-PLY IgG-, control IgG-, and PBS-treated mice infected with *S. pneumoniae *D39 serotype 2**. Histological appearance of representative lungs from mice infected intranasally with *S. pneumoniae *serotype 2 and treated with anti-PLY IgG (A), and control IgG (B). Numerous leukocytes can be seen in the peribronchial and perivascular areas, and considerable vascular distension and hemorrhage take place during the progression of pneumococcal colonization. Lungs of mice treated with anti-PLY IgG reveal alveoli, bronchioles, and vessels structurally normal, with no signs of acute inflammation and lower leukocyte infiltration. Blood vessel (v), alveolar space (a), bronchiole (b). Scale bars 50 μm. (C) Numbers of infiltrating cells were counted in random areas of lung tissue sections H&E-stained. Results are means ± SD of 3 mice and are representative of three independent experiments. *, *P *< 0.05 for a comparison of anti-PLY IgG- treated mice with PBS-treated mice, and +,*P *< 0.05 for a comparison of anti-PLY IgG with control IgG-treated mice, as determined by two-way ANOVA followed by the Bonferroni test.

To determine PLY pro-inflammatory activity, the number of leukocytes was compared in lung tissue of PBS-, control IgG-, and anti-PLY IgG-treated pneumococcus-infected mice (Figure 3). Lungs from anti-PLY IgG-treated mice (Figure 3A) had a lower number of inflammatory cells than control IgG-treated mice (Figure 3B). H&E-stained lung sections from PBS-treated mice resembled those obtained from the control IgG group. Although PBS-treated mice revealed more infiltrating leukocytes than mice treated with control IgG, no statistical differences were found (Figure 3C), even though clinical differences in survival time had previously been shown [26].

## Discussion

In this study, we attempted to explore the relationship between PLY and the lung injury observed during the progression of pneumococcal infection in a mouse intranasal challenge model [26]. In pneumococcal pneumonia, the cytolytic activity of PLY has been implicated in lung colonization, breakdown of the capillary-epithelial barrier, and bloodstream dissemination of the microorganisms. The results of our experiments demonstrate that levels of PLY in BAL are related to the bacterial burden. The significant decrease in number of bacteria after 12 h of infection is probably due to the host response, and was also observed in another intranasal model of pneumococcal pneumonia [28]. PLY expression in lungs has been previously demonstrated by immunofluorescence staining [29]. In CSF of animals with experimental pneumococcal meningitis concentrations of PLY up to 4.34 μg/ml were measured [30], while in human CSF, PLY was detected at concentrations of up to 180 ng/ml [31]. To the best of our knowledge, this is the first report of toxin quantification during experimental pneumococcal pneumonia.

Our findings showed low amounts of PLY quantified either before (< 30 pg/ml) or after pneumococcal lysis (< 1000 pg/ml), which have been shown to be sublytic in various cellular types. PLY is a cholesterol-dependent cytolysin capable of making pores in virtually all cholesterol-containing membranes [4], although it affects distinct cellular types differently [32]. PLY causes half-maximal lysis of endothelial and epithelial cell types at concentrations of approximately 15 HU/ml [33,34]. It was also reported that only very high concentrations of PLY (1 to 20 μg/ml) have cytotoxic effects in alveolar epithelial cells [13,35]. In isolated perfused rat lungs, 100 HU/ml of toxin caused extensive damage to the alveolar epithelium [36]. Recently, it has been shown that application of 0.25 or 2.5 μg of PLY aerosolized or infused into isolated murine lungs, led to impressive vascular leakage and formation of pulmonary edema, while sub-cytolytic PLY doses (0.001–0.1 μg) caused gap formation and moderate generation of stress fibers [37]. Concentrations of 0.1 μg/ml are not cytotoxic for fibroblast [12] or brain microvascular endothelial cells [38], while in ependymal models, 1 μg of PLY caused complete tissue destruction [39]. In general, concentrations of PLY under 10 ng/ml are sublytic, and concentrations necessary for a direct cytotoxic effect of PLY are higher than those causing immunomodulatory or functional interference [31]. The concentrations of free toxin measured in BAL are possibly underestimates of the amounts of PLY released by bacteria, since an unknown portion of the toxin liberated from bacteria probably binds quickly to the lung tissues [31]. PLY amounts in BAL should be only taken into account together with the histological examination of the tissues. In this regard, there was an inverse relation between bacterial load/PLY concentrations and tissue damage. A decrease in CFU, probably due to bacterial lysis, produced an increase in lung injury, possibly due to the released toxin.

The match between PLY- and apoptosis-positive cellular types provides strong support for the pro-apoptotic role of PLY. The marked decrease in apoptotic cells in anti-PLY IgG-treated mice corroborates that PLY has an important role in apoptosis. Although apoptosis in alveolar macrophages has been associated with bacterial internalization [40], programmed cell death directly induced by PLY has been described in alveolar macrophages and nasopharyngeal epithelium [14,41]. *S. pneumoniae *produces two morphologically distinct forms of programmed cell death [15]. We found TUNEL- and caspase-9 positive staining, suggesting that both apoptosis pathways could be induced by pneumococci in lung tissues. Confirmation of this finding could be evaluated by using pneumococci *ply*-negative mutants [7], although isogenic PLY-negative mutants of D39 exhibited slower growth in the lungs, and the maintenance of the same rate of progression of infection would be required to prove the direct effect of the toxin. Moreover, PLY and/or other microbial factors including cell wall components that can trigger induction of apoptosis in the host have been identified [22], and a relation between alveolar macrophage apoptosis and pneumococcal inoculum has been demonstrated [42]. Hence, anti-PLY antibody treatment should only neutralize the pro-apoptotic effects of the toxin and, furthermore, we could not discount the possibility that a decrease in bacterial load could lead to a decrease in cellular apoptosis.

Reports in the literature have suggested that the TUNEL assay detects DNA fragmentation from both necrotic and apoptotic nuclei [43]. In our study, there was no significant difference between TUNEL- and caspase-9 positive cell numbers, suggesting that apoptosis was the major cause of cell death in pneumococcal-infected lung tissues in our model. Although necrosis induced by pneumococci has been observed *in vitro *[13,44], necrosis during non-resolving pneumonia *in vivo *has not been found [45,46].

It has been reported that the interaction of PLY with TLR-4-containing cells, such as macrophages, leukocytes and epithelial cells, mediates apoptosis as a mechanism of host defense against pneumococcal infection [14,47]. In contrast, TLR4 was only protective against a low inoculum in another model of pneumococcal pneumonia [45].

In our pneumococcal pneumonia model, apoptosis of alveolar macrophages, leukocytes and bronchial epithelial cells was not associated with a host benefit, since the inoculum we use is 100% lethal in mice. Pathogen-induced modulation of the host cell-death pathway may eliminate key immune cells or promote evasion of host defences that can limit infection [19]. Apoptosis of resident alveolar macrophages 12 h after infection removes the first line of host defense in innate immunity, and apoptosis of bronchial epithelium after 24 h post-infection eliminates the first physical barrier against pneumococcal dissemination. Leukocyte apoptosis was found in areas of cellular infiltration during pneumococcal infection. Up-regulation of leukocyte genes encoding key effectors of apoptosis is another pathogen-driven mechanism to evade host immunity after phagocytosis of bacteria [48]. Our results strongly suggest that apoptosis removes cells that have a key role in combating the infecting organism, and the consequential effect might be on other aspects of the immune cell function different from reducing inflammation.

Sublytic PLY concentrations and non-staining of the vascular endothelium with anti-PLY antibodies suggest that the pore-forming capability of PLY is not the only agent responsible for damage to vascular endothelial barriers. Hence, the vascular distension that takes place during infection may pave the way for pneumococci to reach the bloodstream, despite the fact that pneumococcal transcytosis through microvascular endothelial cells [49] could also contribute to bloodstream dissemination. The relative contribution of the cytotoxic and proinflammatory capacities of PLY to pulmonary damage has been controversial. The findings from a mouse model of intratracheal challenge using large amounts of PLY (40 ng/mouse) indicated that lung injury resulted from a direct cytotoxic effect of the toxin and was independent of recruited leukocytes [50].

Pneumococci induce the expression of pro-inflammatory and chemotactic cytokines by lung epithelium, thus contributing to leukocyte invasion [35]. It is well documented that PLY induces inflammatory events during pneumococcal pneumonia [1], and the interaction of PLY with host immune cells has been shown to induce the release of inflammatory mediators [8,24,25,47]. Our findings reveal that administration of anti-PLY antibodies produces a marked decrease in inflammation, lung injury, and leukocyte infiltration. The interaction of PLY with TLR4 stimulates the inflammatory response in macrophages independently of the cytolytic properties of the toxin [47]. Mutants lacking the *ply *gene show a decreased infiltration of leukocytes in foci of infection [28]. Exaggerated inflammatory responses mediated by PLY may favor microbial survival by promoting premature, auto-oxidative exhaustion of phagocytes and oxidative dysfunction of B and T lymphocytes [24].

## Conclusion

We have previously demonstrated that passive administration of antibodies to PLY protects mice against pneumococcal pneumonia [26]. Our current findings indicate that the capacity of PLY to trigger inflammatory cell activity could play the major role in inducing the tissue damage that is observed in our model of pneumococcal pneumonia. Taken together, our results indicate that PLY at sublytic concentrations induces lethal inflammation in lung tissues and could be involved in apoptosis of cells of the host immune system, which is important to pathogen survival.

## Competing interests

The author(s) declare that they have no competing interests.

## Authors' contributions

MMGS conceived and designed the study, coordination and manuscript preparation. NF was involved in animal experimentation, tissue sample preparation and toxin quantification. RV participated in animal experimentation. AA was involved in histopathological studies and image analysis. FV participated in coordination of experiments and manuscript preparation. KF was involved in sample preparation and quantification. LAP participated in the design and coordination of experiments and the manuscript preparation. FJM conceived and designed the study and the coordination of experiments. All authors contributed to drafting of the manuscript and approved the final manuscript.
